# Conservative management of delayed presentation of intraperitoneal bladder rupture following caesarean delivery: A case report

**DOI:** 10.1016/j.ijscr.2019.04.050

**Published:** 2019-05-10

**Authors:** Ismaeel Aghaways, Rawa Bapir, Tahir A. Hawrami, Nishtman M. Thahir, Mohammed Abed Al Kadum Hassan, Karzan Mohammed Salih Hassan

**Affiliations:** a University of Sulaymaniyah, Faculty of Medical Sciences, School of Medicine, Department of Surgery, Sulaymaniyah, Kurdistan Region, Iraq; b Sulaymaniyah Surgical Teaching Hospital, Urology Department, Sulaymaniyah, Kurdistan Region, Iraq; c Shaheed Shawkat Haji Musheer Hospital, Urology Department, Said Sadiq/Sulaymaniyah,Kurdistan Region, Iraq; d Soma Private Hospital, Sulaymaniyah, Kurdistan Region, Iraq; e Sulaymaniyah Surgical Teaching Hospital, Department of Surgery, Sulaymaniyah, Kurdistan Region, Iraq

**Keywords:** Cesarean delivery, Conservative management, Case report, Pseudo azotemia, Urinary bladder rupture

## Abstract

**Introduction:**

Bladder injury is an uncommon complication of cesarean delivery (CD) with an incidence ranging from 0.0016% to 0.94%. The risk factors are emergency CDs, subsequent CDs, trial of normal delivery after CD and whether adhesions are present or not. The presentation is either immediate intraoperative event or delayed bladder rupture and urinary ascites.

**Case presentation:**

A 35-year-old female presented with considerable abdominal distension, shortness of breath, oliguria, straining to void and elevated levels of blood urea and serum creatinine 11 days after an uneventful Caesarean delivery. Abdominal ultrasound showed marked ascites. Immediate resuscitation was done, a urethral catheter and percutaneous intraperitoneal pigtail catheter were inserted confirming urinary ascites. Next day cystoscopy was performed and revealed a perforation at the posterior wall of the bladder. She was kept on conservative treatment with adequate urine diversion through both urethral and intraperitoneal catheter. Her condition improved and responded well.

**Discussion:**

To the best of our knowledge this presentation of delayed intraperitoneal bladder rupture post CD is the second case reported in literature. Although surgical repair is regarded as the treatment of choice for intraperitoneal bladder injury, conservative treatment may succeed in properly selected cases. Non-operative managements include indwelling transurethral Foley catheter alone, percutaneous peritoneal drain alone or combined Foley catheter and percutaneous peritoneal drain for complete urinary drainage.

**Conclusion:**

Delayed urinary bladder rupture is a very rare complication of cesarean delivery. Non-operative treatment can be a viable alternative to surgical repair in carefully selected patients.

## Highlights

• Bladder injury is an uncommon complication of cesarean delivery with an incidence ranging from 0.0016% to 0.94%.
• Delayed blabber rupture post CD may present with urinary ascites and elevated serum creatinine mimicking acute kidney injury.
• Cystography is the diagnostic imaging of choice with the reported accuracy of 85%–100%.
• Surgical repair is the treatment of choice for intraperitoneal bladder injury.
• Conservative management with intraperitoneal and urethral catheter may succeed in properly selected cases.

## 1 Introduction

Iatrogenic bladder injury is a reported complication that warrants caution when performing pelvic and/or abdominal retroperitoneal surgeries. Cesarean Delivery (CD) is amongst the procedures that carry low risk for bladder injury, with incidence ranging from 0.0016% to 0.94% [1,2]. Most of the minor bladder injuries heal without consequences, yet significant morbidity could ensue. Identifying risk factors for bladder injury during CDs along with proper post-injury management and follow up bears significant importance. Few studies are found on the subject and some of the risk factors mentioned are emergency CDs, subsequent CDs, trial of normal delivery after CD and whether adhesions are present or not [3]. In accordance to SCARE criteria, we here present a case of delayed presentation of bladder rupture post CD [4].

## 2 Case report

**Fig. 1:**
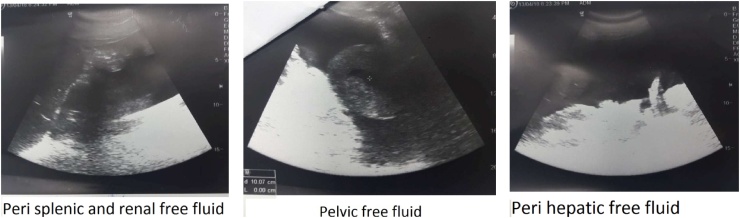
Abdominal and pelvic ultrasound showing marked ascites.

**Fig. 2:**
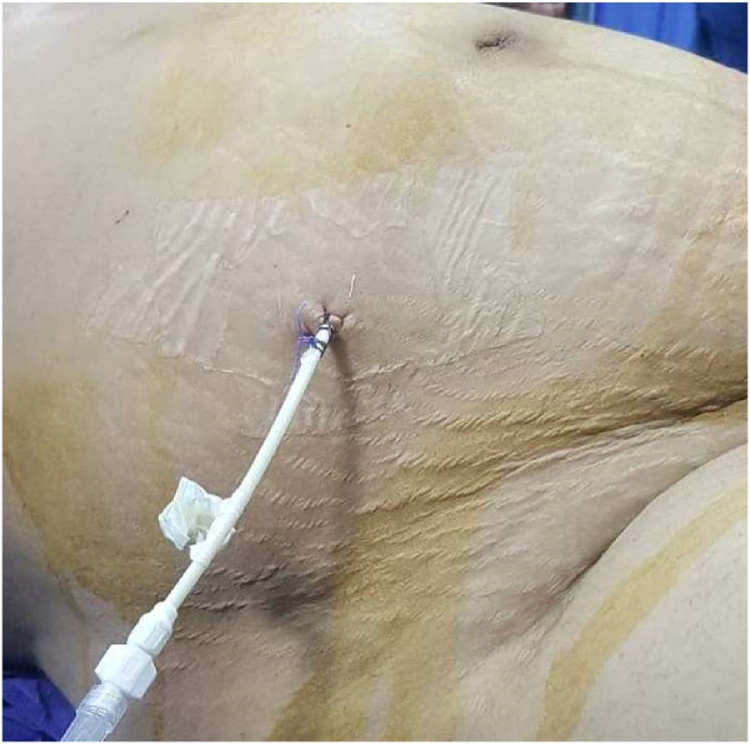
Percutaneous pigtail drain at the right lower abdomen.

**Fig. 3:**
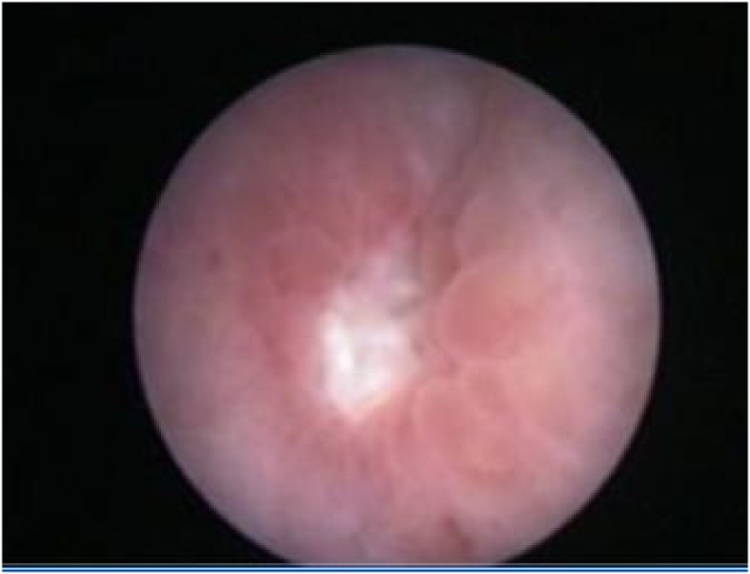
Cystoscopic view of a small perforation at the posterior wall of bladder.

**Fig. 4:**
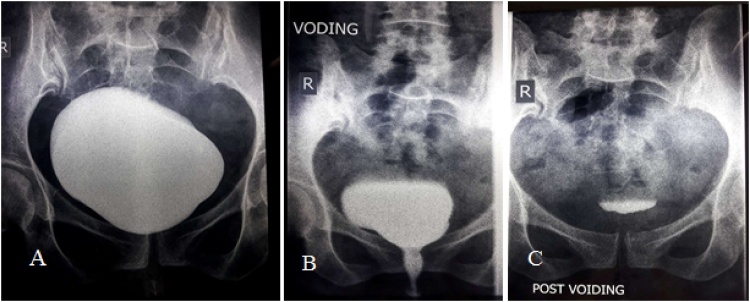
Retrograde cystography showing no contrast extravasation. (A) Filling phase, (B) Voiding (C) Post voiding film.

A 35-year-old healthy pregnant lady with a history of 3 previous cesarean sections was scheduled for her 4th cesarean delivery. The operation was performed under spinal anesthesia. Severe adhesion of the urinary bladder to the lower uterine segment was encountered but there was no apparent lower urinary tract injury. Her postoperative period was uneventful and she was discharged home the next day. On the 11th postoperative day she was readmitted to the emergency unit at 11 pm with considerable abdominal distension, shortness of breath and difficulty of micturition with straining to void. Further questioning revealed sudden inability to void 5 days earlier followed by mild hematuria and passing a small amount of urine. On examination she was dyspneic, the abdomen was distended, pulse rate was 100 BPM, blood pressure was 100/60 mm/Hg and afebrile. Immediate resuscitation was started and Foley catheter was inserted which drained 100 ml concentrated urine. Serum creatinine (6.8 mg/dl), blood urea (123 mg/dl) and serum potassium (5.6 meq/l) were high. Abdominal and pelvic ultrasound showed marked ascites (Fig. 1), normal both kidneys with no hydronephrosis. A trial of diagnostic and therapeutic ascitic fluid drainage was performed by putting a percutaneous 12 French pigtail catheter in the right lower abdomen under ultrasonic guidance (Fig. 2). Six and a half liters of clear fluid was drained. Biochemical investigation of the drained fluid showed high urea (145 mg/dl) and creatinine (20 mg/dl). Diagnosis of urinary ascites was confirmed. Later on, there was a dramatic improvement in the general condition of the patient. Next day blood chemistry was repeated and showed normal blood urea and serum creatinine. Through cystoscopy, we detected a perforation at the posterior wall of the bladder (Fig. 3), while both ureters were normal. Then a Foley catheter was fixed to completely drain the urine in addition to the peritoneal drain to allow the perforation to heal. The patient was put on intravenous fluid and antibiotics with monitoring of vital signs. Three days later the intraperitoneal drain nearly had no drainage and no more free fluid could be seen on ultrasound scan, therefore the tube was removed. The patient was discharged with Foley catheter in place. Two weeks later voiding cystography was performed which showed no more leak (Fig. 4) and the Foley catheter was removed. The patient was discharged home with a good health and returned to her daily life.

## 3 Discussion

Bladder injury is an uncommon complication of cesarean delivery. The mode of presentation is either intraoperative occurrence which is seen in most of the cases or delayed bladder perforation post cesarean delivery. The latter is very rare, in which the patient presents with urinary ascites and elevated serum creatinine due to its reabsorption through the peritoneal membrane, mimicking acute kidney injury. Tai CK et al reported the first and the only case of delayed bladder rupture 14 days after cesarean section. They postulated the mechanism of the perforation to be due to partial mural tear by diathermy or retraction during separation of the bladder from the lower uterine segment. This will lead to the formation of a weak point that may become a full thickness perforation during straining [5]. The same scenario may be the cause of bladder perforation in our case 11 days post procedure and to the best of our knowledge, this is the second case in literature.

The standard imaging technique for the diagnosis of bladder injury is retrograde cystography with a reported accuracy of 85%–100%, but there are still reports of false negative results especially in cases with small perforations and inadequate bladder distension with contrast material. In these cases CT cystogram is an alternative where even a small amount of contrast extravasation can be detected [5]. However in our case none of the aforementioned investigations were done, because they were not available in our hospital at the time of presentation at midnight and the general condition of the patient was unstable, favoring immediate intervention through drainage of the peritoneal cavity under ultrasonic guidance using a 12 Fr pigtail tube. Once we confirmed that the drained fluid is urine, we performed cystoscopy next morning and a perforation was found at the posterior wall of the bladder (Fig. 3).

Although surgical repair is regarded as a standard care for intraperitoneal bladder injury, there are multiple successful trials of nonoperative management in the literature; among them are intraperitoneal bladder perforations during transurethral resection of bladder tumor, spontaneous bladder rupture and bladder rupture due to blunt trauma. Nonoperative management includes indwelling transurethral Foley catheter alone, percutaneous peritoneal drain alone or combined Foley catheter and percutaneous peritoneal drain for complete drainage [[6], [7], [8], [9], [10]]. In the study of Agrusa et. al, where 75 patients with post-operative (open or laparoscopic) complications were managed through second look laparoscopy and achieved 84%% success rate on first trial [11]. The only other reported delayed post CD intraperitoneal bladder rupture by Tai Ck et al was also managed through laparoscopic suturing of the perforation [5]. However in our case, we managed the patient non- operatively through combined Foley catheter and percutaneous intraperitoneal drainage. The time of patient presentation and her dramatic response to the mentioned measures made laparoscopy unnecessary and complete recovery was achieved.

## 4 Conclusion

Delayed urinary bladder rupture is a very rare complication of cesarean delivery. It may present as urinary ascites causing elevated blood urea and serum creatinine mimicking acute kidney injury. Non-operative treatment can be a viable alternative to surgical repair in carefully selected patients.

## Conflicts of interest

There is no conflict of interest.

## Sources of funding

None to be stated.

## Ethical approval

Approval has been given by Ethical committee of University of Sulaymanyiah.

## Consent

Written informed consent was obtained from the patient for publication of this case report and accompanying images. A copy of the written consent is available for review by the Editor-in-Chief of this journal on request.

## Author’s contribution

**Operating surgeos:** Ismaeel Aghaways, Nishtman M. Thahir, Rawa Bapir

**Design and idea**: Ismaeel Aghaways, Rawa Bapir, Tahir A. Hawrami, Nishtman M. Thahir, Mohammed Abed Al Kadum Hassan and Karzan Mohammed Salih Hassan.

**Drafting:** Rawa Bapir, Ismaeel Aghaways and Tahir A. Hawrami

**Data aquision**: Rawa Bapir, Karzan Mohammed Salih Hassan, and Nishtman M. Thahir

**Final revision**: Tahir A. Hawrami, Rawa Bapir, Nishtman M. Thahir, and Karzan Mohammed Salih.

## Registration of research studies

Not applicable.

## Guarantor

The corresponding author is the guarantor of submission.

## Provenance and peer review

Not commissioned, externally peer-reviewed.
